# Dynamic computed tomography manifestations of simulated wooden foreign bodies in blood-saline mixtures with variable concentrations and retention times

**DOI:** 10.1038/s41598-023-35636-0

**Published:** 2023-06-05

**Authors:** Daoming Zhu, Xiaoling Li, Huiyan Zhao, Meng Zhou, Honghao Zhu, Daming Qin, Biyong Tan, Xianzhuo Zhang, Xingrong Hu

**Affiliations:** 1 Department of Radiology, The Central Hospital of Enshi Tujia and Miao Autonomous Prefecture, Enshi, Hubei China; 2 Management Bureau of Guanshuihe National Wetland Park in Xuan’en County, Xuanen, Hubei China; 3 The Second Clinical College of Xinxiang Medical University, Xinxiang, Henan China; 4 The First School of Clinical Medicine, Lanzhou University, Lanzhou, Gansu China

**Keywords:** X-ray tomography, Trauma

## Abstract

Diagnosing wooden foreign bodies (WFBs) using computed tomography (CT) is often missed, leading to adverse outcomes. This study aims to reduce misdiagnoses by exploring the density variation of blood-saline mixtures in ex vivo models. Twenty *Cunninghamia lanceolata* sticks, selected as WFB models, were randomly assigned to five groups: a control group (saline) and four experimental groups immersed in blood-saline mixtures with varying concentrations. The samples were then placed in a constant-temperature water bath at 36.8 °C. CT scans were performed in the lowest and highest density areas, and the volume of the low-density areas was measured at the post-processing workstation. Finally, the effects of time and concentration on imaging were analyzed, and fitting curves were generated. The blood-saline mixture concentration and time significantly affected the CT number in the three areas. WFB images changed dynamically over time, with two typical imaging signs: the bull's-eye sign on the short axis images and the tram line sign on the long axis images. Fitting curves of the CT number in the lowest density areas with different concentrations can quantify imaging changes. The CT number of the lowest density areas increased with time, following a logarithmic function type, while the CT number of the highest density areas exhibited a fast-rising platform type. The volume of the low-density areas decreased over time. The time of damage caused by WFBs and the influence of varying blood and tissue fluid contents at the damaged site should be considered in the diagnosis. Imaging changes from multiple CT scans at different times can aid in diagnosis.

## Introduction

Wooden foreign bodies (WFBs) retained in the body are common clinical emergency cases, leading to an increased rate of mortality and disability. Despite advances in imaging technologies, detecting WFBs remains challenging for clinicians. Radiologists and clinicians conducting initial diagnoses and treatments misdiagnose 38% of patients with foreign bodies^1,2^. Typically, patients undergo evaluations months or even years after the initial injury, but clinical assessments might not reveal a history of skin puncture^1^. In a case reported by Samuthrat et al.^3^, a 2-year-old girl suffered a transoral penetrating brain injury after falling onto a bamboo chopstick, which penetrated her hard palate, fractured the middle skull base, and lacerated and contused the temporal lobe. Nishio et al.^4^ reported a 13-year-old female who fell while holding wooden chopsticks at the age of six, resulting in one becoming lodged in her right eyelid. Although a physician examined her immediately, no symptoms developed for seven years. Their department discovered a brain abscess and an intracranial foreign body using computed tomography (CT) and magnetic resonance imaging (MRI) seven years after the penetrating injury. WFBs have a loose structure, readily absorb water and expand, break into pieces easily, and serve as an excellent culture medium for microorganisms. Moreover, they often carry pathogenic bacteria, chemical irritants, and toxins, which can easily spread to neighboring tissues, causing infection or chemical damage^3,4^.

CT is the most accurate and preferred method for diagnosing WFBs^5–7^. However, WFBs' CT numbers are often similar to the densities of air, fat, soft tissue, blood, and calcified lesions, leading to a high misdiagnosis rate^8–11^. Furthermore, a traditional clinical view suggests that WFBs are not visible on CT scans. If WFBs are overlooked, preoperative qualitative and positioning diagnoses become challenging, resulting in failed operations or the need for repeated surgeries^4^. Therefore, systematically studying the changing imaging characteristics of WFBs is crucial for improving WFB diagnosis and minimizing patient suffering^13^.

WFBs undergo changes upon entering the body, and they typically reside in a blood saline mixture (BSM) environment, making their diagnosis complex and difficult^14^. The composition of BSMs is complex, and the viscosity of BSMs with different concentrations varies^15^. Numerous studies and case reports have focused on WFBs; however, misdiagnosis case reports remain the primary focus^14,16^. Research on the dynamics of the density of retained wooden foreign bodies is still lacking. Thus, investigating these dynamics holds significant clinical diagnostic and practical value.

## Materials and methods

### Study design

An ex vivo model was established, in which WFBs were immersed in BSMs of varying concentrations, simulating the internal environment. Using CT, we obtained the dynamic changes of the CT number for the lowest density area, the highest density area, and the volume of the low-density area of the WFBs at multiple predetermined time points.

**Figure 1:**
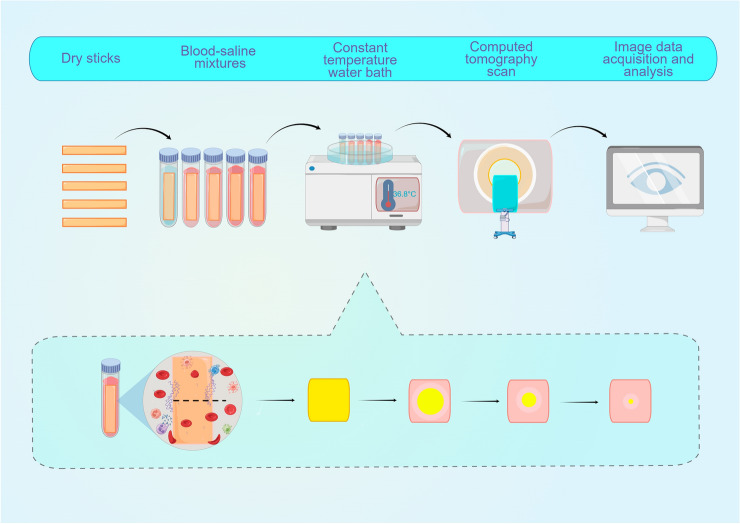
Flowchart of the precedure and changes of wood strips in blood saline mixture.

A 12-year-old artificial *Cunninghamid lanceolata* fast-growing forest in the southwest of Hubei Province, with a diameter of approximately 15 cm, was selected and provided by the School of Forestry and Horticulture, Hubei University for Nationalities. Wood from the same annual ring area on the same layer of the xylem was cut into small wooden sticks measuring 40 mm × 2 mm × 2 mm. The wooden sticks were then dried at 50 °C to constant weight (absolute dry state). Density was calculated based on mass and volume, ρ≈0.38 × 10^3^ kg/m3. Finally, all small sticks with statistically significant differences in length, width, height, and volume were excluded. The procedure's flowchart is shown in Fig. 1.

### Ex vivo blood saline mixtures model

A constant temperature water bath (Jintan Baita Xinbao Instrument Factory) was employed. An appropriate volume of water was placed in the bath, and the temperature was set to 36.8 °C. Plastic tubes with a 5 ml capacity and good sealing were filled with BSMs of different concentrations. The prepared wooden sticks were fixed using thin plastic sheets and suspended in the center of the tubes, respectively. Physiological saline served as the water component of the BSM. Five concentrations were established based on the proportion of whole blood contained in the BSMs, including the control group (saline group), trial (T) group T25 (containing 25% human whole blood), T50 group (50% human whole blood), T75 group (75% human whole blood), and T100 group (100% human whole blood), with four replicates for each group.

### Scanning methods

#### Scanning equipment and parameters

A 64-slice spiral CT scanner was utilized (Philips Ingenuity 64 Slice CT Scanner, Philips, Netherlands). The tube voltage was set to 120 kV, tube current to 320 mA, rotation time to 2 s/cycle, display field of view to 25 mm × 25 mm, reconstruction layer thickness to 1.0 mm, pitch to 0.984, and a standard algorithm (Filtered Back Projection) was applied for reconstruction.

#### Scanning

After establishing the test tube models, they were placed on the CT scanning bed for the initial CT scan to acquire baseline image information. Then, the models were submerged in test tubes containing BSMs of varying concentrations and placed in a constant temperature water bath. At 6-h intervals, each group of test tubes was removed from the water bath, scanned horizontally and vertically to collect data at each time point, and scanned up to 612 h (25.5 days) after model establishment^17^.

#### Image data acquisition and analysis

The raw data were uploaded to the GE ADW4.7 workstation for post-processing analysis. Regions of interest in the highest density areas and lowest density central areas were outlined separately to obtain CT numbers. The workstation's volume measurement software was used to measure the volume of the WFBs' low-density areas, defined as an area with a CT number < 0 HU. After completing data collection for the four group replicates, the mean was calculated and entered into the database for analysis.

#### Statistical analysis

Repeated-measures analysis of variance and least significant difference (LSD) were employed for statistical analysis to explore the differences between the CT numbers of the lowest density areas, the highest density areas, and the volume of the low-density areas of WFBs at different times and BSMs with varying concentrations (*P* = 0.05). An aspherical test was performed on the variables. The differences between BSMs with different concentrations at the same time were tested for significance using a one-way analysis of variance and LSD (*P* = 0.05). All data were subjected to the Shapiro–Wilk normality test before analysis, and data that did not conform to normal distribution were transformed based on log (x + k) or 1/x. SPSS 25.0 software was used for data analysis and fitting, while Origin 2021 software was utilized for graphing.

## Results

### Dynamic image signs of WFBs changing over time

**Figure 2:**
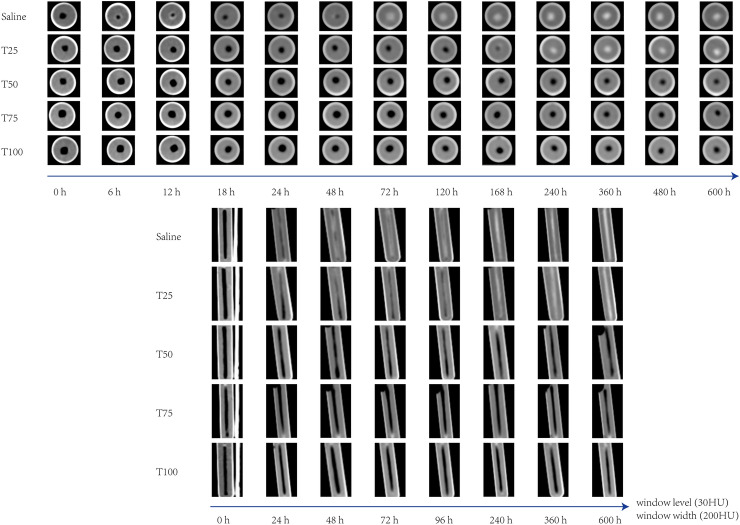
Dynamic image signs of wooden foreign bodies changing over time. The bull's-eye sign on the short axis images and the tram line sign on the long axis images.

In this study, we observed two typical imaging signs by analyzing the CT scan images of WFBs. The first sign was the bull's-eye sign. In the short-axis images, the edges of the WFBs displayed ring-shaped high-density shadows, while the central areas showed round-shaped low-density shadows. Against the background of the high-density areas at the edges, they resembled bull's-eyes (Fig. 2). The second sign was the tram line sign. In the long-axis images of the multi-planar reconstruction, the WFBs exhibited strip-shaped high-density shadows on both edges and low-density shadows in the center, resembling a track (Fig. 2).

### Effects of BSMs with different concentrations on CT number of the lowest density areas of WFBs

**Figure 3:**
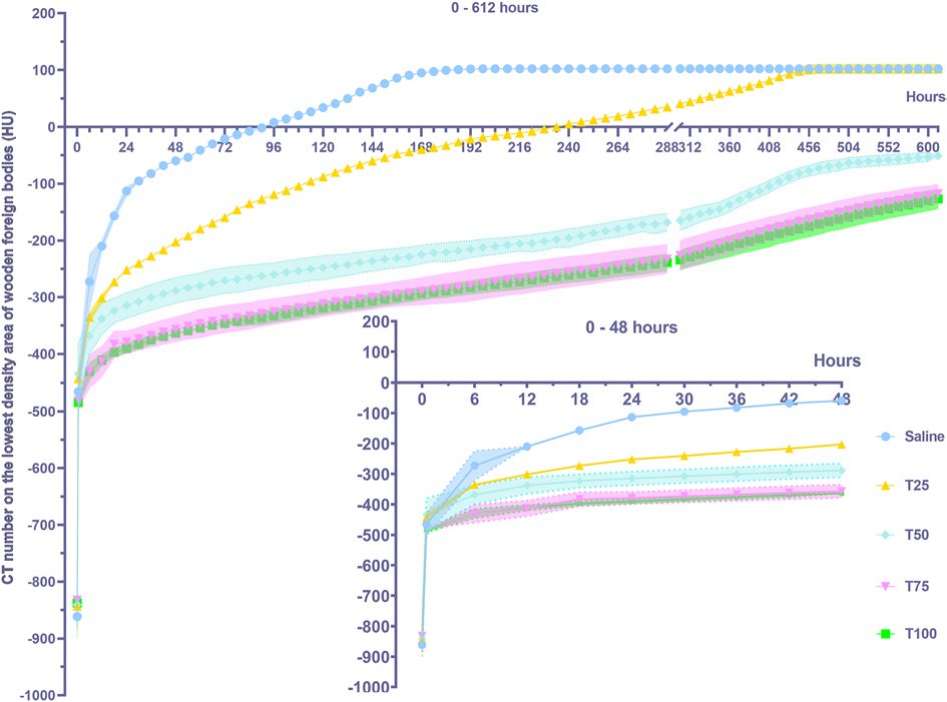
Influence of simulated blood saline mixture concentration on the CT number of the lowest density areas of wooden foreign bodies (mean ± SE, N = 4). Different lowercase letters indicate significant differences in different content. (*P* < 0.05).

Both BSM concentration and time had a highly significant effect on the CT number of the lowest density areas of wooden foreign bodies (*P* < 0.001, Table 1), with a highly significant interaction between them (Table 1, *P* < 0.001). Overall, the increase in CT number in the lowest density areas decreased with increasing BSM concentrations in unit time. The CT number curves of each group of time-lowest density exhibited a rapid rise initially, followed by a slow rise (Fig. 3). Before the experiment began, there was no difference in the CT number of the lowest density areas of the wooden sticks between the groups. After the sticks were immersed in BSMs with different concentrations, the CT number of the lowest density areas of each group increased significantly within the first 6 h, and the CT number of the lowest density areas between the groups showed differences. The CT number of the low-density areas in the Saline group increased the fastest, 589 HU higher than the initial value (− 861.5 HU). The CT number of the lowest density areas in the T100 group increased the slowest, only increasing by 407.5 HU compared with the initial value (− 837.5 HU). Afterward, the changes in CT number in the lowest density areas of each group displayed a slowly increasing trend. Among them, the lowest density areas of the NC group and T25 group disappeared at the 90th and 234th hours, respectively (CT number of WFBs > 0 HU). The fitting curves of different CT numbers of each group over time are shown in Table 2.

**Table 1:** Time, concentration, and the interaction of the two on the CT number of the lowest density area, the highest density area, and the volume of the low density area repeated measurement analysis of variance results.

Factor	Degree of freedom	CT number of the lowest density area (HU)		CT number of the highest density area (HU)		Volume of the low density area (mm^3^)	
		*F*	*P* value	*F*	*P* value	*F*	*P* value
Concentration	4	410.63	< 0.001	5.04	0.009	3228.99	< 0.001
Time	51	5523.68	< 0.001	9041.12	< 0.001	3834.45	< 0.001
Concentration × time	204	81.58	< 0.001	0.22	1.00	203.22	< 0.001

**Table 2:** Fitting curves of CT number of the lowest density areas of each group over time.

Variables	Saline	T25	T50	T75	T100
Model	ExpAssoc	Log3P1	Log3P1	Log3P1	Log3P1
Equation	y = y0 + A1*(1–exp(− x/t1)) + A2*(1–exp(− x/t2))	y = a–b*ln(x + c)	y = a–b*ln(x + c)	y = a–b*ln(x + c)	y = a–b*ln(x + c)
y0 / a	− 847.62484 ± 10.52168	− 562.0078 ± 316.02167	− 535.16267 ± 201.88272	− 587.67282 ± 194.96256	− 589.09241 ± 190.70632
A1 / b	568.85164 ± 12.95004	− 102.90716 ± 59.15695	− 67.07945 ± 37.79097	− 64.21223 ± 36.49557	− 62.94436 ± 35.69883
t1 / c	0.87731 ± 0.06001	0 ± 2.97323	0 ± 2.91385	0 ± 2.93962	0 ± 2.93336
A2	383.82868 ± 7.36488				
t2	60.75528 ± 1.92106				
Reduced Chi-Sqr	442.86378	889,026.8213	362,810.1617	338,363.5422	323,751.1327
R^2^ (COD)	0.9787	0.93938	0.79579	0.78619	0.81884
Adjusted R^2^	0.97842	0.93898	0.79445	0.78479	0.81765

The fitted curve diagrams can be found in the supplementary material.

### Effects of BSMs of different concentrations on CT number of the highest density areas of WFBs

**Figure 4:**
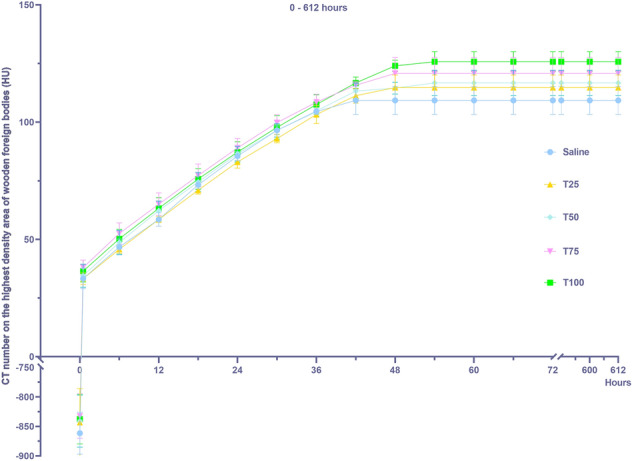
Influence of simulated blood saline mixture concentration on the CT number of the highest density areas of wooden foreign bodies (mean ± SE, N = 4). Different lowercase letters indicate significant differences in different content. (*P* < 0.05).

Both BSM concentration and time significantly affected the CT number of the highest density areas (Table 1, *P* < 0.001). During the experiment, the CT number curves of the time-highest density areas exhibited a fast-rising platform type (Fig. 4). Before the experiment started, the WFBs in each group were all low-density without high-density areas; after being immersed in BSMs, high-density areas appeared at the 6th hour, and the CT number of the highest density areas tended to be constant. The CT numbers of the highest density areas of the five groups ranged between 109.25 HU and 125.75 HU, and the CT numbers of the highest density areas of each group were significantly different.

### Effects of BSMs of different concentrations on the volume of the low-density areas of WFBs

**Figure 5:**
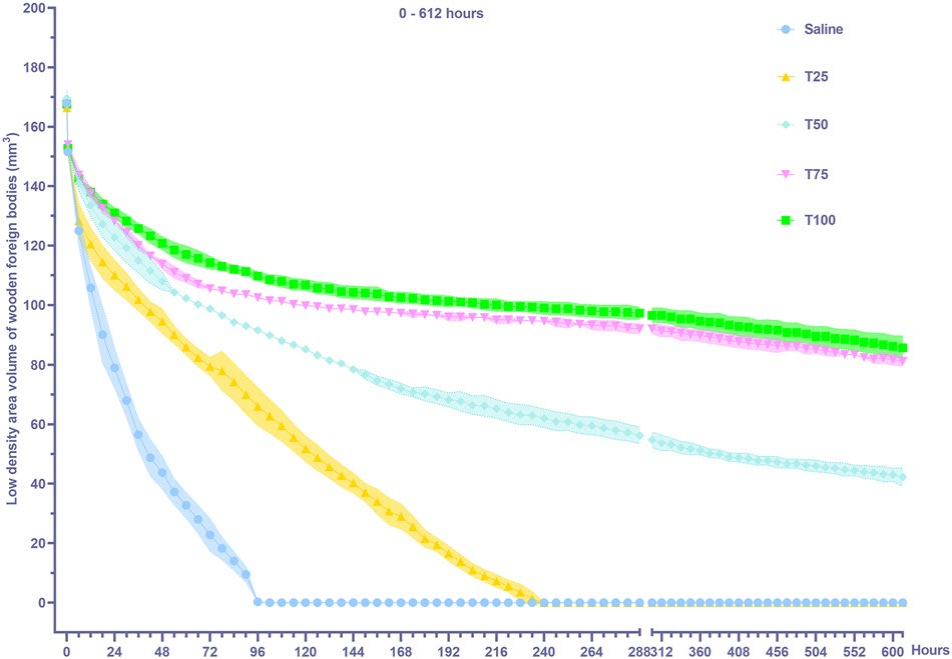
Influence of simulated blood saline mixture concentration on the volume of the low density areas of wooden foreign bodies (mean ± SE, N = 4). Different lowercase letters indicate significant differences in different content. (*P* < 0.05).

Both BSM concentration and time had highly significant effects on the volume of the low-density areas of WFBs (Table 1, *P* < 0.001), with highly significant interactions between them (Table 1, *P* < 0.001). The degree of the decrease in the volume of the low-density areas decreased as the concentrations of the BSM increased. The time and low-density areas volume curves of the Saline group and T25 group exhibited a rapid decline, while the time and low-density areas volume curves of the T50 group, T75 group, and T100 group decreased slowly (Fig. 5). Initially, there was no difference in the volume of the low-density areas among the five groups. After being immersed in the BSMs, the volume of the low-density areas of each group decreased significantly within the first 6 h. The degree of volume reduction in the low-density areas gradually decreased from the Saline group, T25 group, T50 group, T75 group, to the T100 group. Compared with the initial value (167.85 mm3), the volume of the low-density areas in the Saline group was reduced by 42.85 mm3; the volume of the low-density areas in the T100 group was only reduced by 25.09 mm3 from the initial value (167.59 mm3). Among them, the volume of the low-density areas disappeared in the Saline group at the 96th hour and the T25 group at the 240th hour (CT number of WFBs > 0 HU). Ultimately, the remaining groups still had low-density areas of varying sizes.

## Discussion

### Principal findings

This study demonstrates that WFB images change dynamically over time, exhibiting two typical imaging signs: the bull's-eye sign on short-axis images and the tram line sign on long-axis images. We developed fitting curves of CT numbers in the lowest density areas across different concentrations to quantify the imaging changes of WFBs. Both the BSM concentration and time have a significant effect on the CT number of the lowest density areas of WFBs, with a notable interaction between them. The CT number of the lowest density areas increases logarithmically over time; however, this increase diminishes as BSM concentrations rise. Both the BSM concentration and time have a significant effect on the CT number of the highest density areas of WFBs. The CT number curves display a fast-rising platform type. The volume of the low-density areas decreases over time, with the rate of decrease lessening as BSM concentrations increase.

### Potential mechanisms

#### The effect of the wood’s water absorption capacity on density changes

WFB density, which determines the CT number, is closely related to the water absorption capacity of wood materials^18^. This capacity primarily depends on the cellulose and pore structure of wood, as confirmed by Gao^19^. Within the first 6 h of this study, the CT number of the lowest and highest density areas of each group increased rapidly. This increase can be attributed to the large internal cell cavity pores of the wood during the initial stage of water absorption, which rely on the capillary system composed of axial tracheids to absorb water with the help of capillary force, while lignocellulose quickly absorbs water^20,21^.

Moreover, it has been reported that the isotherm adsorption process of wood mainly involves rapid monolayer adsorption on the adsorption sites within the wood. This process may represent another significant reason for the swift density changes of WFBs within the first 6 h^21^. As relative humidity increases, second-layer and multi-layer adsorption gradually appear, resulting in more water molecules being adsorbed by WFBs and a corresponding increase in density^22,23^. As the water content of lignocellulose rises, its hydrophobicity also increases, causing the water absorption capacity to slow down^20^. The water absorption process of WFBs progresses gradually from the surface to the center; the further from the wood surface, the slower the water absorption rate, and the relatively slower the density change. Consequently, the density of the area near the wood surface of the WFB changes most rapidly, whereas the density of the central area changes more slowly (Figs. 1, 2).

#### Effects of BSMs of different concentrations on the density changes of WFBs

The change in WFB density essentially represents a process in which the air within the wood gap, under the absolute dry state of the WFB, is continuously replaced by free water and other substances from the surrounding environment. Due to the different concentrations of BSMs, free water enters WFBs at varying speeds, which delays the rate of density changes^24,25^. Simultaneously, as BSM concentration increases, so does the viscosity of the medium, hindering the diffusion of water molecules and reducing the speed at which various molecules from the WFBs' surrounding environment enter the interior, thus delaying the density change rate of the WFBs^26,27^. Additionally, the complex composition of BSMs means that organic macromolecules and various cells in the blood may adhere to the surface, forming a barrier film that slows down the entry of water molecules into the WFBs^28^. Another study has indicated that the gas within the wood foreign matter and the water molecules entering it form a gas–liquid plane, which is not conducive to the direct exchange of water molecules and gas^29^.

### Image characteristics of changes in the density of WFBs and for best practice

As the edge of the WFB is in direct contact with the surrounding environment, the air inside it is replaced at a significantly higher speed than the interior. Consequently, high-density areas appear at the edges while the interior remains low-density. The tram line sign is generally observed on the long-axis of the planar reconstructed image, as seen in the WFBs cases reported by Mertel et al.^30^. The bull's-eye sign is mostly visible on short-axis images, as demonstrated in the cases reported by Peterson et al.^1^. When imaging a WFB, it is essential to consider the patient's description and the position within the image to make an accurate diagnosis.

In this study, the CT number of WFBs in the body varies considerably over time. The initial CT number of WFBs upon entering the body ranges from − 800 HU to − 900 HU, with a density close to that of gas, necessitating differentiation from gas accumulation within the body. As WFBs remain in the body for longer periods, their edge density increases while the central area remains low-density, and the CT number of the low central area gradually increases until the WFB becomes high-density overall^30^. The CT number of the WFB progressively rises to − 100 HU to − 200 HU, at which point the density is close to that of fat, requiring differentiation from fatty tissue. After some time, the density of WFBs will be similar to that of water, soft tissue, blood, or calcification, necessitating differentiation from these counterparts^31–35^. WFBs typically exhibit rapid changes at the edge and slower changes in the central area, resulting in a ring-shaped high-density image. This feature can be used as a characteristic manifestation. Radiologists can calculate the CT number of wooden foreign bodies in different tissues based on the fitting curve equations of time and various concentrations to reduce the likelihood of missed diagnoses.

### Strengths and Limitations

This study presents a novel approach for investigating WFBs by considering the dry state as the initial state and dynamically observing the imaging manifestations and change patterns of WFBs. Additionally, we discussed potential factors influencing the imaging changes of WFBs and elucidated the inherent nature of these changes. This work contributes to a better understanding of WFB imaging manifestations and alterations. Unlike previous studies on WFBs, which were conducted either in vitro or in animals and examined at a single time point^6,7,35^, our research offers a more comprehensive perspective on WFB imaging. This is particularly important given the continuous changes in density and imaging appearance of WFBs in the body.

However, this study has several limitations. First, we only investigated Chinese fir materials, and the CT number changes might differ for various types of wood foreign bodies. Second, we focused on a low-density wood, and it remains to be determined whether high-density wood foreign bodies exhibit similar patterns. Finally, the composition of the organizational microenvironment in the human body is more complex than BSM environment. Future research should aim to establish models to better replicate the in vivo environment. Despite these limitations, our study represents a significant advancement in the field and provides valuable insights for future investigations.

## Conclusion

In summary, this study elucidates the influence of blood concentration and retention time on the density of WFBs within the BSMs. The findings provide valuable insights for the identification and differentiation of WFBs in clinical scenarios. Accurate diagnosis of WFBs necessitates a comprehensive evaluation of the patient's medical history, the surrounding BSM environment, image positioning, and temporal changes.

For cases where WFB retention is suspected, a follow-up scan after a certain period may be recommended. Characteristic alterations in the images from the two scans can facilitate more precise clinical diagnoses. Future investigations should focus on high-quality, well-designed studies and trials that compare multiple diagnostic tools to further enhance the understanding and management of WFB-related cases in clinical practice.

## Supplementary Information

Supplementary Information.

## Data Availability

The datasets used and/or analyzed during the current study available from the corresponding author on reasonable request.

## Abbreviations

WFBs: Wooden foreign bodies
CT: Computed tomography
LSD: Least significant difference
BSM: Blood saline mixture
MRI: Magnetic resonance imaging

## References

[1] Peterson JJ, Bancroft LW, Kransdorf MJ (2002). Wooden foreign bodies: Imaging appearance. AJR Am. J. Roentgenol..

[2] Anderson MA, Newmeyer WL, Kilgore ES (1982). Diagnosis and treatment of retained foreign bodies in the hand. Am. J. Surg..

[3] Samuthrat T, Ye K, Tong Y (2017). Transoral intracranial injury via middle skull base by a blunt chopstick in a child. World Neurosurg..

[4] Nishio Y, Hayashi N, Hamada H, Hirashima Y, Endo S (2004). A case of delayed brain abscess due to a retained intracranial wooden foreign body: A case report and review of the last 20 years. Acta Neurochir. (Wien)..

[5] Covert DJ, Henry CR, Sheth BP (2009). Well-tolerated intracorneal wood foreign body of 40-year duration. Cornea.

[6] Zhao Y, Yang Z, Quan J (2019). Sonographic diagnosis of perforation of the gastric antrum caused by a foreign body: A case report. Medicine (Baltimore).

[7] Arey ML, Mootha VV, Whittemore AR (2007). Computed tomography in the diagnosis of occult open-globe injuries. Ophthalmology.

[8] Lagalla R, Manfrè L, Caronia A (2000). Plain film, CT and MRI sensibility in the evaluation of intraorbital foreign bodies in an in vitro model of the orbit and in pig eyes. Eur.Radiol..

[9] Ober CP, Jones JC, Larson MM (2008). Comparison of ultrasound, computed tomography, and magnetic resonance imaging in detection of acute wooden foreign bodies in the canine manus. Vet. Radiol. Ultrasound..

[10] Adesanya OO, Dawkins DM (2007). Intraorbital wooden foreign body (IOFB): Mimicking air on CT. Emerg. Radiol..

[11] Specht CS, Varga JH, Jalali MM (1992). Orbitocranial wooden foreign body diagnosed by magnetic resonance imaging. Dry wood can be isodense with air and orbital fat by computed tomography. Surv. Ophthalmol..

[12] Ablett M, Kusumawidjaja D (2009). Appearance of wooden foreign body on CT scan. Emerg. Med. J..

[13] Pyhtinen J, Ilkko E, Lähde S (1995). Wooden foreign bodies in CT. Case reports and experimental studies. Acta Radiol..

[14] Gonullu ME, Filinte GT, Cardak NG (2016). The surgical strategy for the intraorbital foreign bodies. J. Craniofac. Surg..

[15] Liu D, Al SE (2002). Retained orbital wooden foreign body: A surgical technique and rationale. Ophthalmology.

[16] Koyama J, Azumi M, Mori T (2020). Microsurgical confirmation of parenchymal contamination of hair in a pediatric patient with a penetrating head injury. Childs Nerv. Syst..

[17] Corzo O, Bracho N (2007). Water effective diffusion coefficient of sardine sheets during osmotic dehydration at different brine concentrations and temperatures. J. Food Eng..

[18] Feng L, He X, Chen J (2018). Complications in transorbital penetrating injury by bamboo branch: A case report. Medicine (Baltimore).

[19] GB/T 1934.1–2009, Method for determination of the water absorption of wood. Available at: http://openstd.samr.gov.cn/bzgk/gb/newGbInfo?hcno=C3E8C9F67AF4B6F0312031205CDB67C1

[20] Xu F, Liang W (1989). A study on water absorption and swelling of 20 kinds of wood in Guangxi. J. Guangxi Agric. Biol. Sci..

[21] Gao Y (2019). Study on the changes and its mechanism of moisture adsorption and absorption properties of high temperature heat treated Chinese fir wood. Chin. Acad. For..

[22] Zauer M, Hempel S, Pfriem A (2014). Investigations of the pore-size distribution of wood in the dry and wet state by means of mercury intrusion porosimetry. Wood Sci. Technol..

[23] Zauer M, Meissner F, Plagge R (2016). Capillary pore-size distribution and equilibrium moisture content of wood determined by means of pressure plate technique. Holzforschung.

[24] Olek W, Bonarski JT (2014). Effects of thermal modification on wood ultrastructure analyzed with crystallographic texture. Holzforschung.

[25] Olek Wiesław, Majka Jerzy (2013). Sorption isotherms of thermally modified wood. Holzforschung.

[26] Moya R, Muñoz F, Jeremic D (2009). Visual identification, physical properties, ash composition, and water diffusion of wetwood in Gmelina arborea. Can. J. For. Res..

[27] Kamiya Y, Takahashi F (2010). Effect of water on permeation, diffusion, and solution of gases in cellophane. J. Appl. Polymer..

[28] Rampp M, Buttersack C, Lüdemann HD (2000). c, T-dependence of the viscosity and the self-diffusion coefficients in some aqueous carbohydrate solutions. Carbohydr. Res..

[29] Franck JM, Scott JA, Han S (2013). Nonlinear scaling of surface water diffusion with bulk water viscosity of crowded solutions. J. Am. Chem. Soc..

[30] Yu XM, Qi CH, Wang CL (2018). Enhancement of water self-diffusion at super-hydrophilic surface with ordered water. Chin. Phys. B.

[31] Ortony JH, Cheng CY, Franck JM (2011). Probing the hydration water diffusion of macromolecular surfaces and interfaces[J]. New J. Phys..

[32] Boncoeur-Martel MP, Adenis JP, Rulfi JY (2001). CT appearances of chronically retained wooden intraorbital foreign bodies. Neuroradiology.

[33] Herman M, Válková Z (1993). Intraorbital wood foreign bodies. Radiology.

[34] Healy JF (1980). Computed tomography of a cranial wooden foreign body. J. Comput. Assist. Tomogr..

[35] Li J, Zhou LP, Jin J (2016). Clinical diagnosis and treatment of intraorbital wooden foreign bodies. Chin. J. Traumatol..

[36] Graham RM, Smyth KL, Langton SG (2008). Intraorbital wooden foreign body. J. Oral. Maxillofac. Surg..

[37] Ginsburg MJ, Ellis GL, Flom LL (1990). Detection of soft-tissue foreign bodies by plain radiography, xerography, computed tomography, and ultrasonography. Ann. Emerg. Med..

