# Supplementary material for: Dynamic computed tomography manifestations of simulated wooden foreign bodies in blood-saline mixtures with variable concentrations and retention times
Source: Sci Rep. 2023 Jun 5;13:9101. doi: 10.1038/s41598-023-35636-0 (PMC10241894; doi:10.1038/s41598-023-35636-0)
Supplement: Supplementary file 1 — Supplementary Information. [file 41598_2023_35636_MOESM1_ESM.docx]

**Supplementary information**

Supplementary figures legends

**
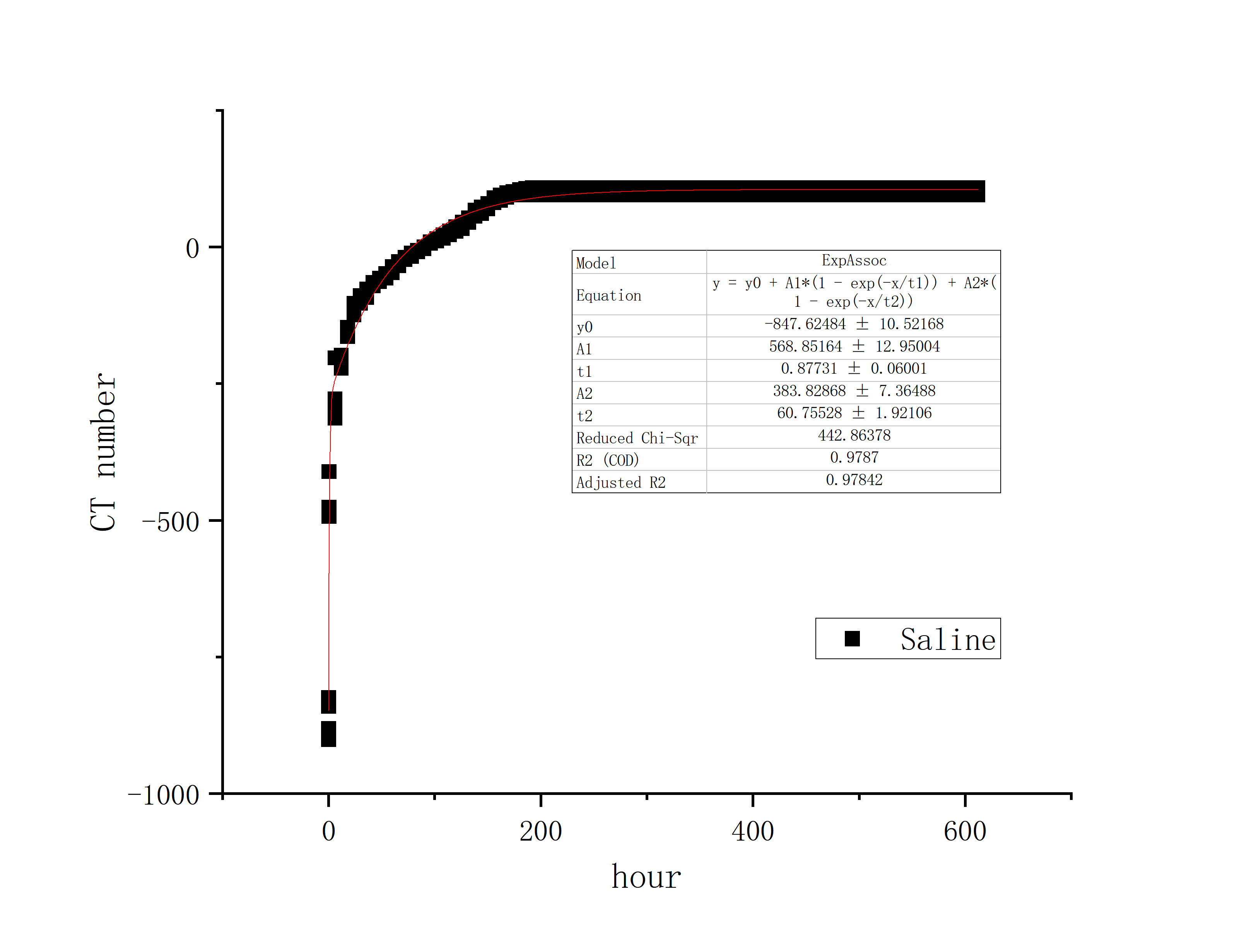
**

**Figure S1.** The fitted curve diagram of CT number of the lowest density areas of saline group (control group).


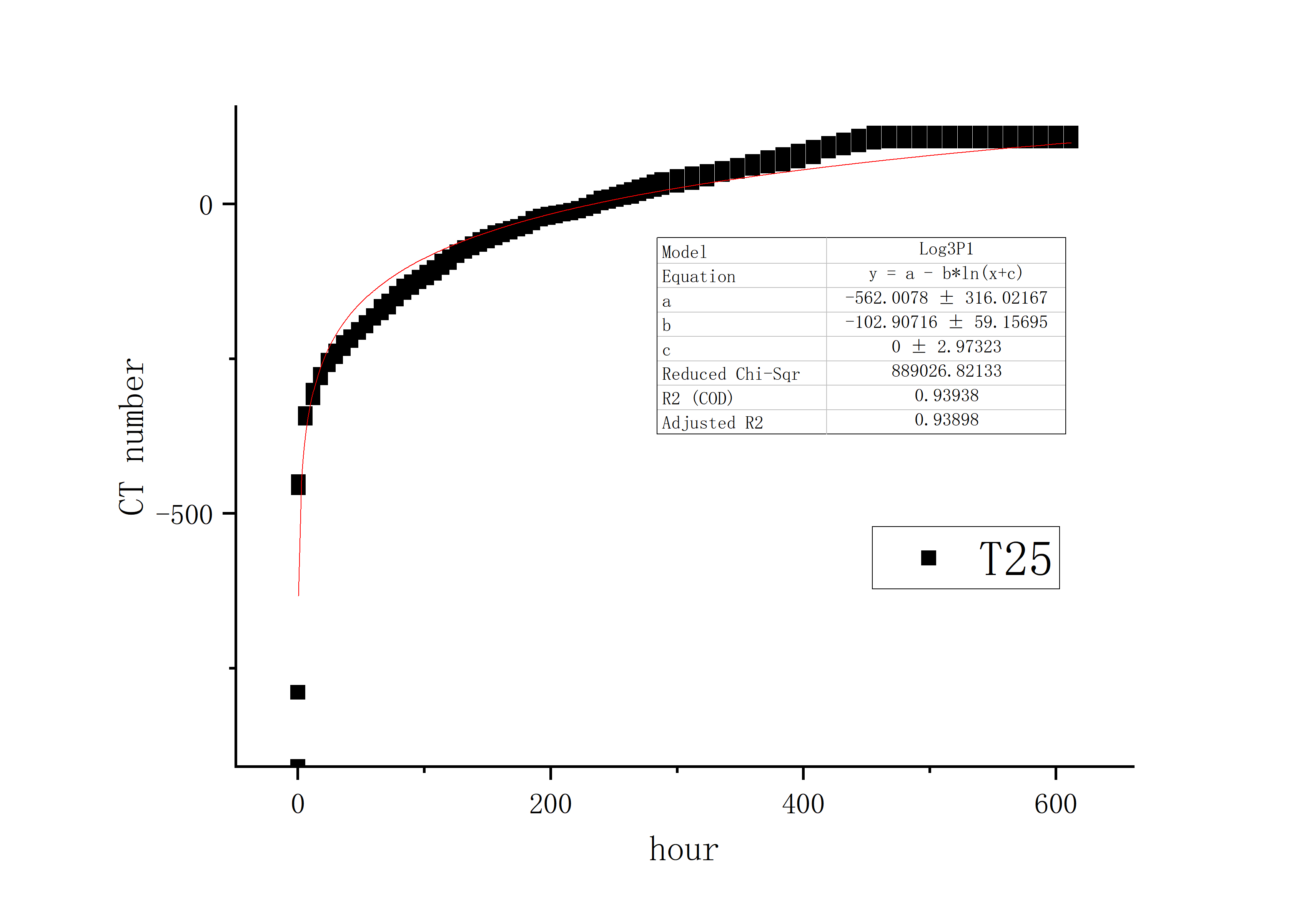


**Figure S2.** The fitted curve diagram of CT number of the lowest density areas of T25 group (containing 25% human whole blood).


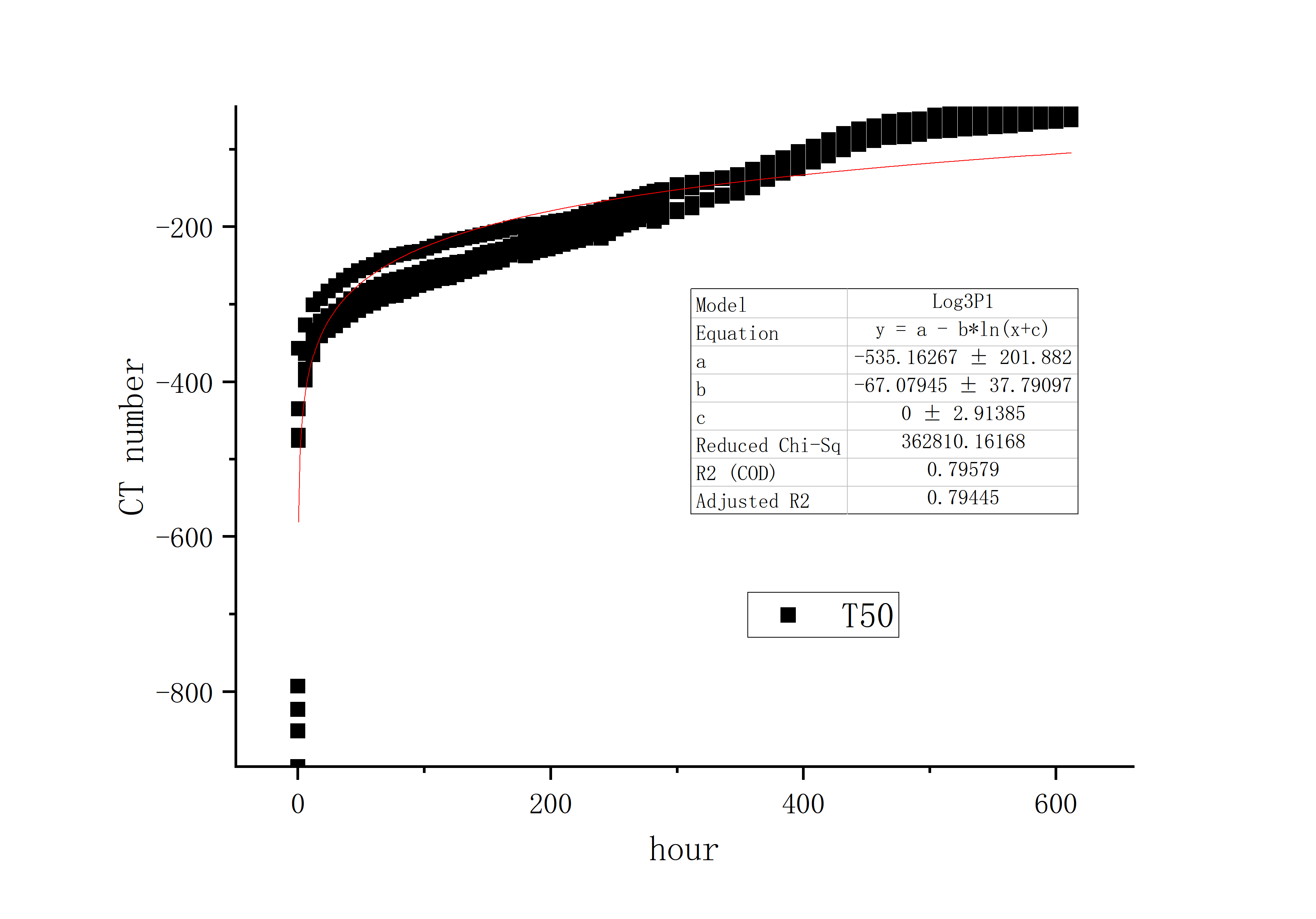


**Figure S3.** The fitted curve diagram of CT number of the lowest density areas of T50 group (50% human whole blood).


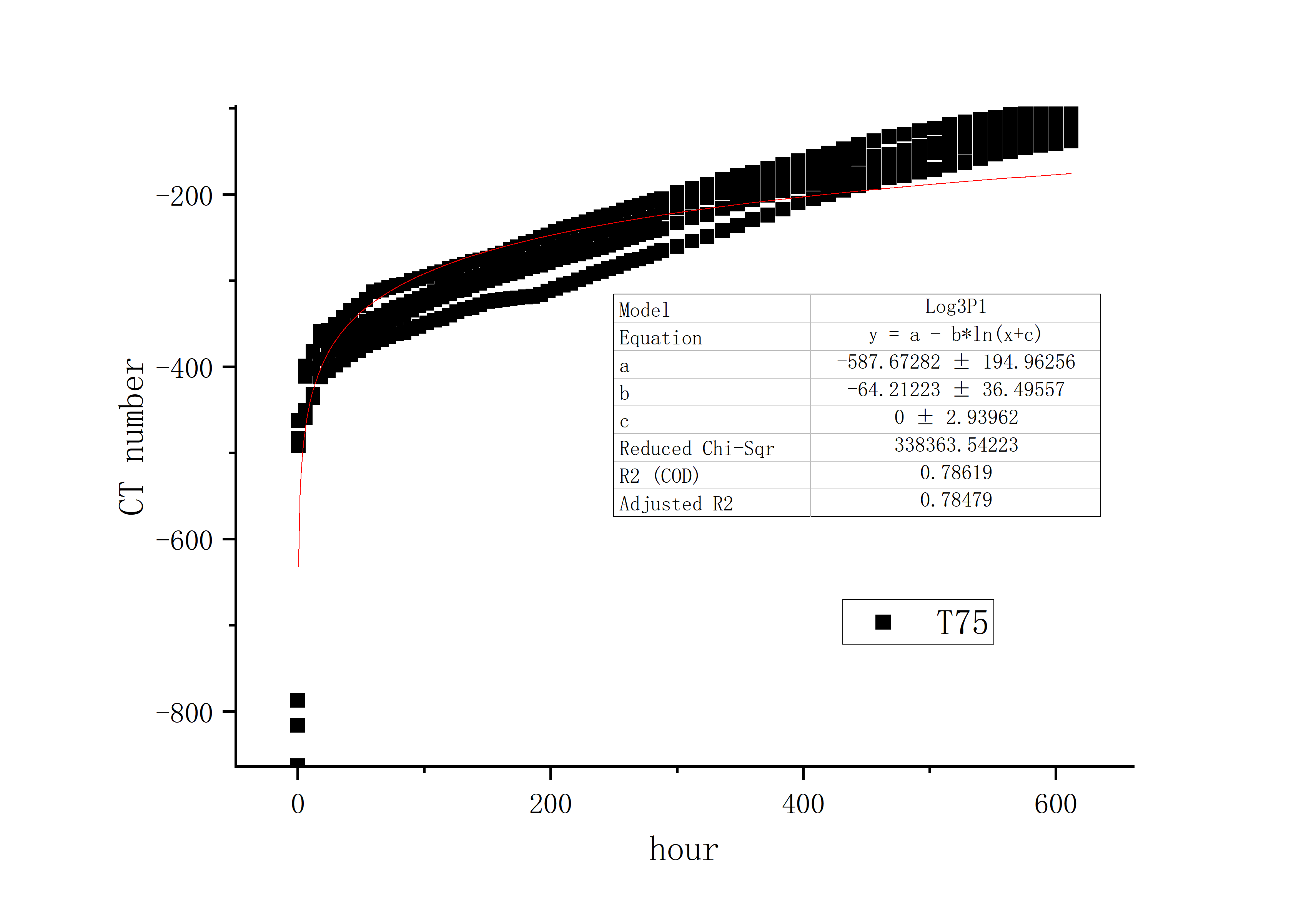


**Figure S4.** The fitted curve diagram of CT number of the lowest density areas of T75 group (75% human whole blood).


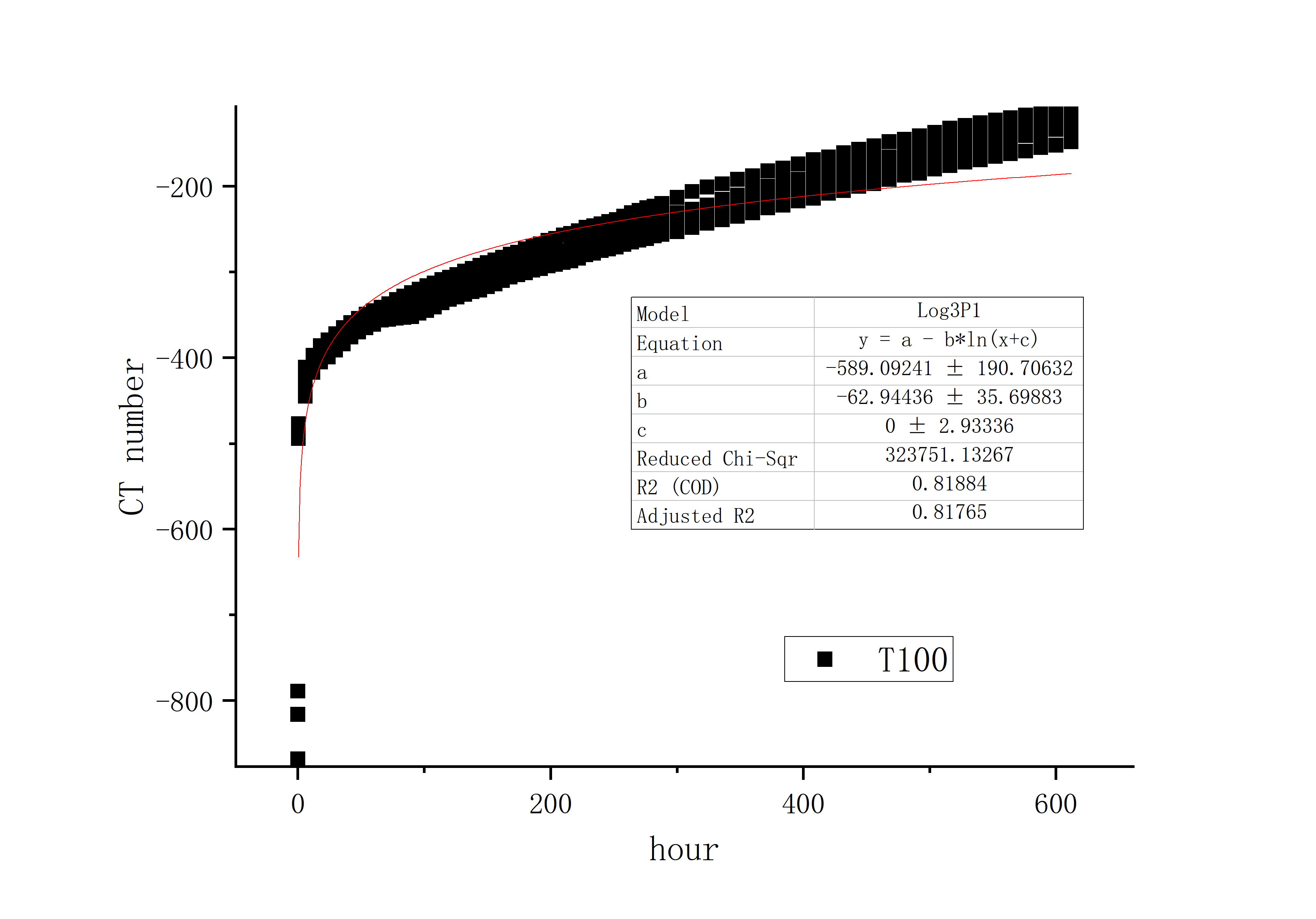


**Figure S5.** The fitted curve diagram of CT number of the lowest density areas of T100 group (100% human whole blood).
